# Eosinophil counts can be a predictive marker of immune checkpoint inhibitor-induced secondary adrenal insufficiency: a retrospective cohort study

**DOI:** 10.1038/s41598-022-05400-x

**Published:** 2022-01-25

**Authors:** Shinobu Takayasu, Satoru Mizushiri, Yutaka Watanuki, Satoshi Yamagata, Mari Usutani, Yuki Nakada, Yuko Asari, Shingo Murasawa, Kazunori Kageyama, Makoto Daimon

**Affiliations:** 1 Department of Endocrinology and Metabolism, Hirosaki University Graduate School of Medicine and Hospital, 5 Zaifu-cho, Hirosaki, Aomori 036-8562 Japan; 2 Present Address: Department of Endocrinology and Metabolism, Sapporo Nijuyonken Hospital, 4-7-20 Nijo Nijuyonken, Nishi-Ku, Sapporo, Hokkaido 063-0802 Japan

**Keywords:** Biomarkers, Endocrinology, Medical research, Oncology, Risk factors, Signs and symptoms

## Abstract

Immune checkpoint inhibitors (ICIs) treatment can result in endocrine immune-related adverse events (irAEs), including pituitary dysfunction. Quick diagnosis of secondary adrenal insufficiency (AI) is challenging because no universal definition of ICI-induced secondary AI has been agreed. The aim of this study was to clarify the clinical features of ICI-induced secondary AI that can be used for screening in standard clinical practice. This retrospective study was performed using the medical records of patients who received ICIs at Hirosaki University Hospital between 1 September 2014 and 31 January 2021. Longitudinal clinical data of patients who developed AI were analyzed and compared with the data of thyroid irAEs. Regression analysis showed a significant correlation between ICI-induced secondary AI and absolute or relative eosinophil counts at pre-onset of AI, as well as differences or rate of increase in eosinophil counts at baseline and at pre-onset. Absolute eosinophil counts > 198.36/µL or relative eosinophil counts > 5.6% at pre-onset, and a difference of 65.25/µL or a rate of eosinophil count increase of 1.97 between the baseline and at pre-onset showed the best sensitivity and specificity. This is the first report to demonstrate that eosinophil counts can be a predictor of ICI-induced secondary AI.

## Introduction

Immune checkpoint inhibitors (ICIs) including antibodies against cytotoxic T-lymphocyte-associated protein 4 (CTLA-4), programmed cell death-1 (PD-1), and programmed death-ligand 1 (PD-L1) have been widely used for advanced malignancies in recent years. However, ICIs can cause immune-related adverse events (irAEs). In endocrine organs, the thyroid and pituitary glands are commonly affected by irAEs^1^. It is important to be aware of the differences in irAEs depending on the type of ICI administered. Anti-PD1/-PD-L1 antibodies rarely instigate pituitary irAEs; however, they can cause (usually isolated) adrenocorticotropin (ACTH) deficiency, which leads to a reduction in the secretion of cortisol and results in adrenal insufficiency (AI)^2,3^. Pituitary irAEs including ACTH deficiency were found to be more frequent with anti-CTLA-4 antibodies and less common with anti-PD-1/PD-L1 antibodies^4^, and patients who received combination therapy of anti-CTLA-4 and anti-PD-1 antibodies were significantly more likely to develop pituitary irAEs^2,4^.

AI may lead to serious and life-threatening consequences without accurate diagnosis and treatment. In ICI-treated patients, AI is suspected on the basis of non-specific symptoms, signs, and/or the results of standard clinical practice because no universal definition of ICI-induced secondary AI exists. The Endocrine Society Clinical Practice Guidelines^5^ recommend diagnostic testing to exclude AI in acutely ill patients with unexplained symptoms suggestive of AI (volume depletion, hypotension, fever, abdominal pain, or hypoglycemia). The clinical practice guidelines of the Japan Endocrine Society^6^ recommend suspecting AI in patients who have the following symptoms: (1) general fatigue and general weakness; (2) appetite loss and weight loss; (3) gastrointestinal symptoms (nausea, vomiting, constipation, and abdominal pain); (4) hypotension; (5) mental disorder (apathy, lethargy, anxiety, and character change); (6) fever; (7) hypoglycemic symptoms; and (8) arthralgia. However, they may be overlooked because ICI-treated patients have advanced malignancies. Hormonal tests are crucial for the diagnosis of AI but rapid assays for cortisol and ACTH are currently not available at most medical institutes; in addition, the cut-off values for the diagnosis of AI by basal cortisol level in serum are variable^5^. As a result, diagnosis of AI may be delayed, which results in life-threatening adrenal crisis^7^. Therefore, the purpose of this study was to develop a prediction marker of ICI-induced secondary AI that relies on routine clinical practice.

## Results

### Patient characteristics

A total of 525 patients who started ICIs were enrolled in this study. The characteristics of the patients are presented in Table 1. Nineteen patients (3.6%) were diagnosed with pituitary irAEs defined as secondary AI. One of these patients was accompanied by thyrotropin and gonadotropin deficiency, and the others were defined as isolated ACTH deficiency. Thirty-four (6.5%) patients with thyroid irAEs were referred to our department, and insulin-deficient diabetes mellitus was observed in two (0.4%) patients. Patients who received PD-1 antibodies (3.4%) or PD-L1 antibodies (1.0%) were less likely to show secondary AI than those receiving ipilimumab or combination therapy (11.4%), as shown in Table 2. Patients receiving ipilimumab or combination therapy (median, 83 days) had a shorter secondary AI diagnosis time compared with those receiving anti-PD1 antibodies (median, 198 days). Most of the patients were aware of their AI-related symptoms earlier than this; however, they presented their symptoms at the date of periodic medical check-up. The median time from onset (when the patients were aware of AI symptoms) to diagnosis was 23 days.

**Table 1:** Characteristics of the overall cohort.

	n = 525
Age (y)	67.1 ± 10.0
**Sex (n)**	
Male	394
Female	131
**Primary sites (n)**	
Lung cancer	263 (50.1%)
Renal-urinary cancer	117 (22.3%)
Head and neck cancer	45 (8.6%)
Malignant melanoma	43 (8.2%)
Gastric cancer	37 (7.0%)
Esophageal cancer	10 (1.9%)
Others	10 (1.9%)
**Endocrine-related irAEs (n)**	
Thyroid irAEs	34 (6.5%)
Secondary adrenal insufficiency	19 (3.6%)
Diabetes mellitus	2 (0.4%)
Others	0 (0.0%)

Age is expressed as average (± standard deviation) at the first administration of immune checkpoint inhibitors.

Abbreviation: *irAEs* immune-related adverse events.

**Table 2:** Clinical course of patients with pituitary AI.

ICI class	ICI	n	AI (n)	AI for each ICI class (n)	Day of pre-onset visit (d)	Day of symptom onset (d)	Day of diagnosis of AI (d)
PD-1	Niv	216	5	13 (3.4%)	164	184	198
	Pem	164	8				
	Niv > Pem	2	0				
PD-L1	Atez	60	0	1 (1.0%)	5	33	72
	Avel	7	0				
	Dur	29	1				
	Dur > Atez	1	0				
PD-1 > PD-L1	Niv > Atez	1	0	0 (0.0%)	0	0	0
	Pem > Atez	1	0				
CTLA-4 or CTLA-4 + PD-1	Ipi or Ipi + PD-1	44	5	5 (11.4%)	62	64	83

A > B indicates change of ICI from A to B. ‘Day’ is the median number of days from the first administration of ICI.

Abbreviation: *AI* adrenal insufficiency, *Atez* atezolizumab, *Avel* avelumab, *CTLA-4* cytotoxic T-lymphocyte-associated protein 4, *Dur* durvalumab, *ICI* immune checkpoint inhibitor, *Ipi* ipilimumab, *Niv* nivolumab, *PD-1* programmed cell death-1, *PD-L1* programmed death-ligand 1, *Pem* pembrolizumab.

To predict ICI-induced secondary AI before onset, we compared a thyroid irAE group, which comprised patients who showed only thyroid irAEs among endocrine-related irAEs. We excluded patients who had shown myelosuppression because of chemotherapy. Patients who received glucocorticoids before diagnosis were also excluded. Thus, 17 and 22 patients were recruited to the ICI-induced secondary AI group (AI group) and the thyroid irAE group, respectively. The characteristics of each group are presented in Table 3 and Supplemental Table 1.

**Table 3:** Characteristics of patients with secondary AI.

	n = 17
Age (y)	67.1 ± 9.8
**Sex (n)**	
Male	12
Female	5
**Primary sites (n)**	
Renal-urinary cancer	8 (47.1%)
Lung cancer	6 (35.3%)
Malignant melanoma	3 (17.6%)
**ICI class (n)**	
PD-1	
Pem	6
Niv	5
PD-L1	
Dur	1
CTLA-4 + PD-1	
Ipi + Niv	4
Pem > Ipi	1
Day of pre-onset visit (d)	119 (163.7)
Day of symptom onset (d)	125 (176.5)
Day of diagnosis of AI (d)	140 (201.9)

Age is expressed as average ± standard deviation at diagnosis. Pem > Ipi indicates change of immune checkpoint inhibitor from pembrolizumab to ipilimumab. ‘Day’ is expressed as the median (average) number of days from the first administration of ICI.

Abbreviation: *CTLA-4* cytotoxic T-lymphocyte-associated protein 4, *Dur* durvalumab, *ICI* immune checkpoint inhibitor, *Ipi* ipilimumab, *Niv* nivolumab, *PD-1* programmed cell death-1, *PD-L1* programmed death-ligand 1, *Pem* pembrolizumab.

### Changes in laboratory findings from baseline to irAEs

**Figure 1:**
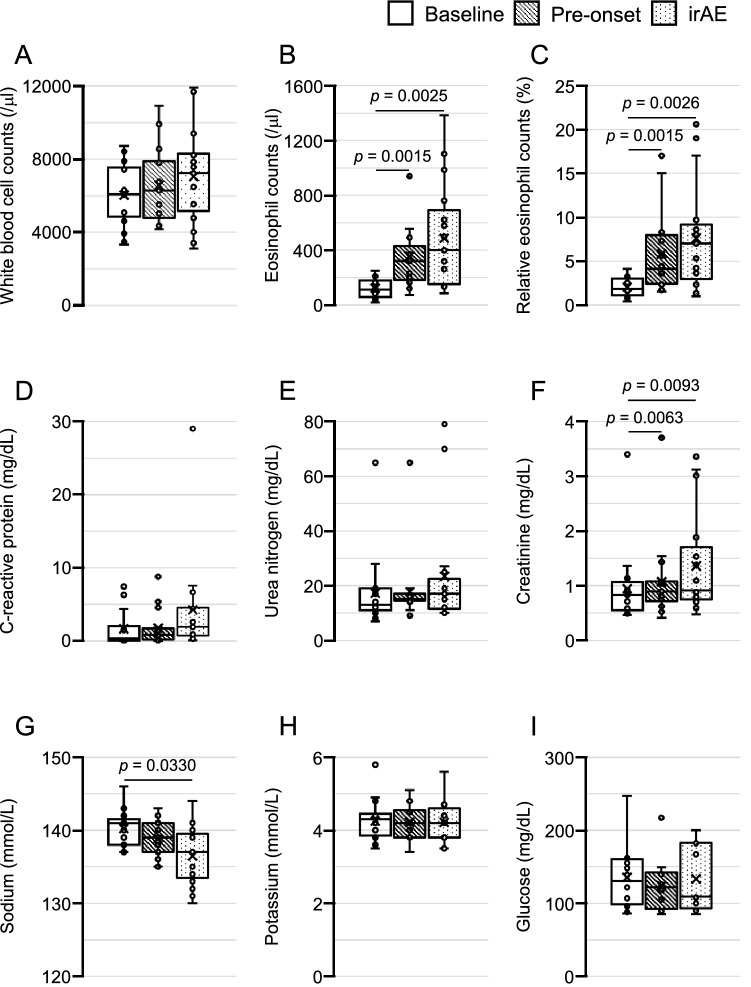
Longitudinal data in the AI group are shown by box plots. (**A**) White blood cell counts, (**B**) absolute eosinophil counts, (**C**) relative eosinophil counts, (**D**) C-reactive protein levels, (**E**) serum urea nitrogen levels, (**F**) serum creatinine levels, (**G**), serum sodium levels, (**H**) serum potassium levels, and (**I**) plasma glucose levels. ‘Baseline’ represents data before the first administration of ICIs. ‘Pre-onset’ represents data checked-up just before the patients experienced AI symptoms. ‘irAE’ represents data at the diagnosis of ICI-induced secondary AI. Statistical analyses were performed by Friedman’s test corrected by Bonferroni’s method. Significant differences of *p* < 0.05 were indicated.

We statistically analyzed the longitudinal results of eight factors (white blood cell count, absolute or relative eosinophil counts, CRP, serum creatinine, serum sodium, serum potassium, and Glc) in routine clinical practice including the factors that are known to be related to AI^5,6^. The results are shown in Fig. 1: ‘baseline’ represents data just before starting ICIs; ‘pre-onset’ represents data checked-up just before patients felt AI symptoms in the AI group; and ‘irAE’ represents data at diagnosis of ICI-induced secondary AI in the AI group and at diagnosis of thyrotoxicosis or hypothyroidism in the thyroid irAE group.

Absolute and relative eosinophil counts, levels of serum sodium, and levels of serum creatinine at irAE in the AI group had significantly changed compared with the baseline (Fig. 1). Absolute eosinophil counts (median/average: 111.0/120.8 at baseline, 319.8/355.1 at pre-onset, and 398.2/486.2/µL at AI), relative eosinophil counts (median/average: 1.8/2.0 at baseline, 4.1/5.8 at pre-onset, and 7.0%/7.6% at AI) and serum creatinine (median/average: 0.8/0.9 at baseline, 0.9/1.1 at pre-onset, and 0.9/1.4 mg/dL at AI) had increased even by pre-onset, whereas this was not the case for the other factors (Fig. 1).

In comparison with the AI group, no factor had changed from the period baseline to irAEs in the thyroid irAE group (Supplemental Fig. 1). No difference was observed when the thyroid irAE group was further divided into two groups according to thyrotoxicosis and hypothyroidism (data not shown).

### Laboratory findings for AI-predicting factors

**Figure 2:**
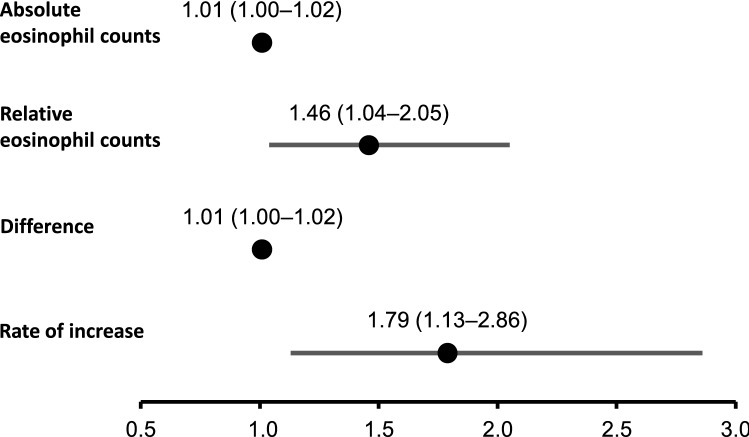
Eosinophil counts as predictors of AI determined by logistic regression analysis. Odds ratios (ORs) with 95% confidence intervals (CIs) are shown. Adjusted for multiple factors: serum sodium, C-reactive protein, and plasma glucose. Difference: difference in absolute eosinophil counts between the baseline and at pre-onset. Rate of increase: rate of increase in eosinophil counts between the baseline and at pre-onset.

**Figure 3:**
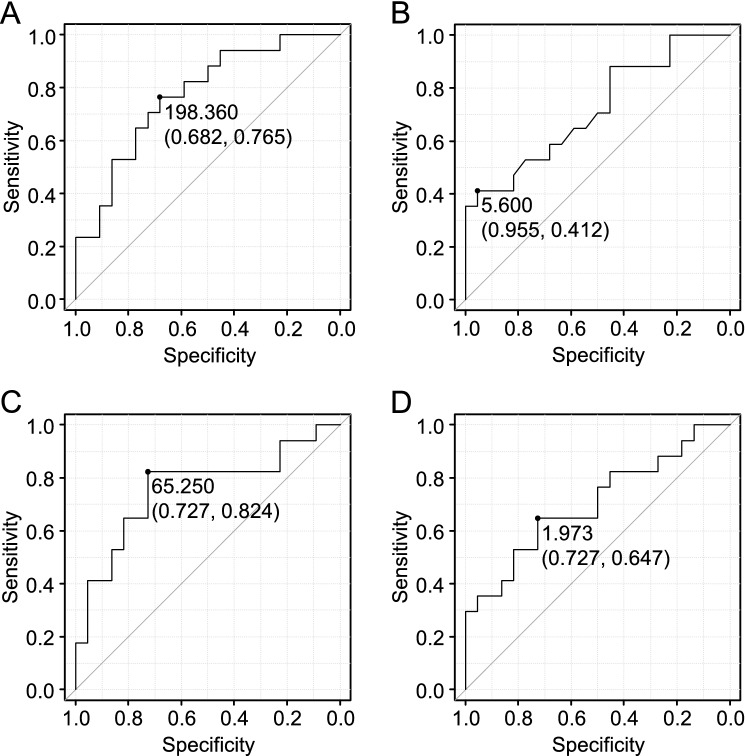
Receiver operating characteristic curves for determination of the cut-off values of the eosinophil counts to predict AI. (**A**) Absolute eosinophil counts, (**B**) relative eosinophil counts, (**C**) difference in absolute eosinophil counts between the baseline and at pre-onset, and (**D**) rate of increase in eosinophil counts between the baseline and at pre-onset.

To develop a prediction marker for ICI-induced secondary AI, we first compared the data at pre-onset in the AI group and the data at irAEs in the thyroid irAE group. The results are shown in Figs. 2 and 3. While no factors had changed at baseline between both groups, absolute and relative eosinophil counts showed significant differences between the two phases of the groups. Then, sodium, CRP, and Glc were included in the multivariate logistic models because the factors are known to be related to AI or adrenal crisis^5,6,8^. Absolute (odds ratio [OR]: 1.01, 95% confidence interval [CI]: 1.00–1.02) and relative (OR: 1.46, 95% CI: 1.04–2.05) eosinophil counts were the only factors that correlated with ICI-induced secondary AI development (Fig. 2). ROC analyses revealed an absolute eosinophil count of 198.36/µL (sensitivity, 76.5%; specificity, 68.2%; area under the curve, 0.77; Fig. 3A) and a relative eosinophil count of 5.6% (sensitivity, 41.2%; specificity, 95.5%; area under the curve, 0.72; Fig. 3B) as optimal cut-off values for determining the risk subjects for AI.

To improve the diagnostic accuracy of the prediction model, we calculated the difference (difference) or rate of increase (rate) of eosinophil counts between the baseline and at pre-onset and re-analyzed the data. The multivariate logistic models that included sodium, CRP, and Glc showed the best correlation between ICI-induced secondary AI development and the ‘difference’ (OR: 1.01, 95% CI: 1.00–1.02; Fig. 2). The ‘rate’ (OR: 1.79, 95% CI: 1.13–2.86) was revealed as the only factor that correlated with ICI-induced AI (Fig. 2). ROC analyses showed that a ‘difference’ of 65.25/µL (sensitivity, 82.4%; specificity, 72.8%; area under the curve, 0.76; Fig. 3C) and a ‘rate’ of 1.97 (sensitivity, 64.7%; specificity, 72.8%; area under the curve, 0.70; Fig. 3D) as optimal cut-off values.

## Discussion

Little information is available on new cases of AI regarding biochemical data obtained before onset of symptoms. Therefore, determining a prediction model for AI is challenging. The purpose of this study was to identify a prediction marker for ICI-induced secondary AI development by biochemical examination. We investigated patients treated with ICIs because they had received periodic medical examination, and analyzed data that were taken before symptom onset. The average and median period between pre-onset visit and symptom onset were 12.8 and 14 days, respectively. We found that eosinophil counts were significantly associated with AI.

Non-endocrinologists usually administer ICIs, and the physician should suspect AI according to the presence of non-specific symptoms such as weakness, fatigue, weight loss, and gastrointestinal symptoms^5,6^. These symptoms may be overlooked in ICI-treated conditions because they are commonly observed in patients with advanced malignancy^4,9^. The signs of ICI-induced AI are based on glucocorticoid deficiency and the resultant hyponatremia, changes in blood count (anemia, eosinophilia, lymphocytosis), and hypoglycemia^5,6^, which can be assessed by routine biochemical practice. Measurement of plasma ACTH and serum cortisol are recommended for AI-suspected patients^5,6^ because transient ACTH elevation occurring prior to the onset of a pituitary irAE has been reported^10,11^. However, rapid assays for cortisol and ACTH are not available at most medical institutes. As a result, diagnosis and treatment is delayed, which can result in progression to a life-threatening adrenal crisis^5,7,8^. Recently, Tahir et al. reported that plasma anti-guanine nucleotide-binding protein G(olf) subunit alpha and anti-integral membrane protein 2B antibodies correlated with ICI-induced hypophysitis^12^, while Kanie et al. reported that serum anti-pituitary antibodies or anti-corticotroph antibodies were associated with the autoimmunity against pituitary hormones or corticotroph^13^. Furthermore, Inaba et al.^14^ and Yano et al.^15^ reported susceptible alleles of human leucocyte antigen in patients with ICI-induced pituitary dysfunction. Although these autoantibodies and human leucocyte antigen may be predictive biomarkers of pituitary irAEs, they are uncommon, and the results were validated in a small number of patients. Therefore, a prediction marker that is commonly and rapidly available for AI is needed.

Patients with pituitary irAEs often present with hyponatremia, defined as a serum sodium level of < 135 mmol/L and a detection rate of 40–100%^10,16–18^. It is also observed in adrenal crisis, the detection rate using the lower limit of the reference ranges from 9 to 50%^8,19–21^. Katabami et al. developed a prediction model for adrenal crisis using data from the same individuals with chronic AI in a stable period during glucocorticoid replacement therapy as a control. They showed that serum sodium and CRP in combination may be feasible for adrenal crisis^8^. We did not define the levels of serum sodium and CRP as prediction factors in the current study. Of 17 patients in our study, 4 showed hyponatremia at diagnosis, while none of the patients showed serum sodium levels of < 135 mmol/L at pre-onset. CRP has been shown to be associated with several tumors^22–25^. We analyzed data from ICI-treated patients who have advanced malignancies; therefore, increased CRP levels at baseline might not make a difference. Cho et al. revealed that hyponatremia could be the first manifestation to indicate AI, followed by other symptoms or physical findings^17^. They performed early diagnosis of ICI-induced AI. Two of four patients showed hyponatremia just before AI diagnosis. However, the levels of neither ACTH nor cortisol were measured before the detection of hyponatremia, and the period between hyponatremia detection and AI symptoms was 4 days. Then, they were diagnosed with AI within almost 1 week. Decreasing ACTH and cortisol levels followed by decreasing sodium and symptom onset can occur consecutively; thus, development of pituitary irAEs may be quick and thus be strongly suspected if serum sodium levels have already decreased.

Although significant differences in the levels of serum creatinine were not shown between the data at pre-onset in AI and the data at irAEs in the thyroid irAE group, the levels of serum creatinine at irAEs or pre-onset had slightly but significantly changed compared with the baseline in the AI group. Of 17 patients, 8 (47.1%) who developed AI had renal-urinary cancer, whereas 6 of 22 patients (27.2%) who developed thyroid irAEs suffered from renal-urinary malignancies. The levels of creatinine in patients who had renal-urinary malignancies seemed to be increasing compared with the remaining patients (data not shown) by AI diagnosis. The progress of renal dysfunction during this period could have affected the results.

It has been recognized for more than 70 years that patients with Addison disease have eosinophilia^26^. Beishuizen et al. reported that 80% of suspected clinical cases of relative adrenal insufficiency had relative eosinophil counts of at least 3%^27^, and some reports have shown that eosinophilia is relevant to adrenal crisis^11,16,28^. Two reports found that eosinophil counts tended to increase before the diagnosis of ICI-induced AI^11,16^. Therefore, we statistically analyzed whether eosinophil counts can predict AI.

We excluded hypereosinophilia or hypereosinophilic syndrome, which is defined as a persistent elevated eosinophil count of ≥ 1500/µL^29^. Of 17 patients, 2 each showed an absolute eosinophil count of > 500/µL at pre-onset AI and > 1000/µL at AI diagnosis. None of the patients showed an eosinophil count of over 1500/µL in this study.

Some studies reported that eosinophilia (absolute eosinophil count > 500/µL) was induced by treatment with ICIs^30–32^. Bernard-Tessier et al. showed immune-related eosinophilia with an estimated frequency of 2.9% and a median peak eosinophil count of 1000/µL^30^; however, they did not report the AI incidence rate of the patients. Scanvion et al.^31^ showed 37 cases of ICI-induced eosinophilia with a median peak eosinophil count of 2700/µL. Three of these patients developed hypophysitis. Furthermore, the AI incidence rate in this study seemed to be higher compared with the incidence of pituitary irAEs in a meta-analysis^2^. Ten of these patients were treated with glucocorticoids for eosinophilia; thus, accurate diagnosis of AI might be difficult and more patients with AI might exist because AI signs and symptoms could be masked by glucocorticoids.

Several studies suggested that eosinophil counts can be a useful predictive marker for irAEs. Diehl et al.^33^ showed an association between irAEs of grade ≥ 2 and higher absolute eosinophil counts (cutoff value = 100/µL); however, they did not report a correlation between eosinophil counts and each type of irAE. Nakamura et al.^34^ showed that both increased baseline eosinophil counts (cutoff value = 240/µL) and relative count (cutoff value = 3.2%) at 1 month after starting ICIs was correlated with endocrine irAEs. They analyzed 45 patients and 56 overall irAEs, which is a relatively small number of subjects. Among 14 patients who developed endocrine irAEs in their study, 10 patients with thyroid dysfunction, 2 patients with pituitary dysfunction, 1 patient with primary adrenal insufficiency, and 1 patient with type 1 diabetes mellitus were presented and were not analyzed separately.

To develop a specific prediction marker for ICI-induced secondary AI, we compared the pre-onset data in the AI group and the data at irAEs in the thyroid irAE group. We chose patients with thyroid irAEs for comparison because thyroid irAEs are the most common among endocrine irAEs^4^. We demonstrated that an absolute eosinophil count of 198.36/µL or a relative eosinophil count of 5.6%, and a difference of 65.25/µL or an increased eosinophil count rate of 1.97 between the baseline and at pre-onset had better sensitivity and specificity for ICI-induced AI screening in the standard examination.

Recently, the association between eosinophil counts and clinical outcome of ICI-treated patients has been reported, and Simon et al. showed that absolute and/or relative eosinophil counts were associated with a better clinical outcome in patients with various cancers^35^. The cut off value of the absolute and relative counts were variable, and they did not report the AI incidence rate in their studies. Kobayashi et al. reported that ICI-induced pituitary irAEs were associated with better overall survival in malignant melanoma and non-small cell lung carcinoma in their prospective study^36^. However, they did not show whether the eosinophil counts had changed during this period. Patients who developed AI with eosinophilia may show better clinical outcomes, which should be further explored in prospective studies.

The strengths of this study are that it provides real-world data focused on the identification of a simple diagnostic marker for AI using standard biochemical examinations. To the best of our knowledge, no clinical studies have focused on the change in eosinophil counts at pre-symptom onset. In addition, we excluded factors that may influence eosinophil counts, that is, myelosuppression, glucocorticoid use, infection, allergy, and drug reaction. This enabled an accurate diagnosis of AI. There are some limitations of our study, including its retrospective design and the relatively small AI population, which results in a lack of power. We used the thyroid irAE group as a control, and thus this is not real case–control comparison. AI incidence and the period between pre-onset and AI diagnosis might be underestimated because ACTH and cortisol were not measured in all patients at all visits.

In conclusion, this is the first report to demonstrate that eosinophil counts can be a predictor of AI due to irAEs. We propose a protocol for the assessment of ICI-treated patients to predict AI, as follows. (1) Check absolute/relative eosinophil counts at all visits and if absolute or relative eosinophil counts are more than 198.4/µL or 5.6% respectively, or the ‘difference’ or ‘rate’ of the eosinophil counts at the baseline and at pre-onset are more than 65.3/µL or 2.0 respectively, exclude other causes of increased eosinophils. (2) Measure ACTH and cortisol, then inform the patients to be aware of the possibility of AI symptom development and visit as soon as possible at onset. (3) When transient ACTH elevation is confirmed at a previous visit, future AI development may be suspected. (4) Diagnose AI accurately and treat sufficiently. (5) Repeat (2) and (3) if eosinophil counts further increase. This strategy can greatly assist early diagnosis and the timing of referral to an endocrinologist. Further investigation in a prospective study is necessary for validation of the protocol and the cut-off values.

## Methods

This retrospective cohort study was performed using the medical records of patients who started ICI at Hirosaki University Hospital between 1 September 2014 and 31 January 2021. The follow-up period of each patient was at least 3 months.

### Ethics approval and consent to participate

This study was approved by the Institutional Review Board and Ethics Committee of the Hirosaki University Graduate School of Medicine (approval number, 2021-075), and was conducted in accordance with the principles of the Declaration of Helsinki. Informed consent was waived by the Institutional Review Board and Ethics Committee of the Hirosaki University Graduate School of Medicine as retrospective study.

### Consent for publication

We provided each patient with the opportunity to opt out of the study using our website.

### Assessments

ACTH and cortisol were tested before starting ICI; thereafter, patients with symptoms suggestive of AI were tested, while other patients were tested at the discretion of the treating physician. ACTH deficiency was first suspected according to low levels of ACTH and/or cortisol. Then, the patients were referred to our department, and secondary AI was defined according to the Japan Endocrine Society clinical guidelines^6^. The cut-off for diagnosis of ACTH deficiency was decreased peak serum cortisol value (< 18 μg/dL) and/or impaired responses of ACTH (< twofold of baseline) in corticotropin-releasing hormone loading tests or insulin tolerance tests. Then the date of diagnosis of the secondary AI was defined as the day on which low levels of ACTH and/or cortisol were confirmed.

### Statistical analysis

Comparisons of longitudinal data were performed by Friedman's test and corrected by Bonferroni's method. The statistical significance of the difference in clinical data values between two groups (nonparametric) was assessed by Mann–Whitney U test. Univariable logistic analysis were performed with factors that are known to be related to AI. Then, sodium, C-reactive protein (CRP), and plasma glucose (Glc) were included to calculate the correlation of eosinophil counts with AI in the multivariate logistic models. Receiver operating characteristic (ROC) curve analysis was performed to determine a cut-off value of the eosinophil counts to predict AI. All analyses were performed using JMP pro version 15.0 (SAS Institute Japan Ltd., Tokyo, Japan) or EZR version 1.54 (Saitama Medical Center, Jichi Medical University, Saitama, Japan), which is a graphical user interface for R (The R Foundation for Statistical Computing, Vienna, Austria).

## Supplementary Information

Supplementary Information.

## Data Availability

Data are available on reasonable request.

## Abbreviations

ACTH: Adrenocorticotropin
AI: Adrenal insufficiency
CI: Confidence interval
CTLA-4: Cytotoxic T-lymphocyte-associated protein 4
ICIs: Immune checkpoint inhibitors
irAEs: Immune-related adverse events
OR: Odds ratio
PD-1: Programmed cell death-1
PD-L1: Programmed death-ligand 1
ROC: Receiver operating characteristic

## References

[1] Michot JM, Bigenwald C, Champiat S, Collins M, Carbonnel F, Postel-Vinay S (2016). Immune-related adverse events with immune checkpoint blockade: A comprehensive review. Eur. J. Cancer.

[2] Barroso-Sousa R, Barry WT, Garrido-Castro AC, Hodi FS, Min L, Krop IE (2018). Incidence of endocrine dysfunction following the use of different immune checkpoint inhibitor regimens: A systematic review and meta-analysis. JAMA Oncol..

[3] Faje A, Reynolds K, Zubiri L, Lawrence D, Cohen JV, Sullivan RJ (2019). Hypophysitis secondary to nivolumab and pembrolizumab is a clinical entity distinct from ipilimumabassociated hypophysitis. Eur. J. Endocrinol..

[4] Stelmachowska-Banaś M, Czajka-Oraniec I (2020). Management of endocrine immune-related adverse events of immune checkpoint inhibitors: An updated review. Endocr. Connect..

[5] Bornstein SR, Allolio B, Arlt W, Barthel A, Don-Wauchope A, Hammer GD (2016). Diagnosis and treatment of primary adrenal insufficiency: An endocrine society clinical practice guideline. J. Clin. Endocrinol. Metab..

[6] Yanase T, Tajima T, Katabami T, Iwasaki Y, Tanahashi Y, Sugawara A (2016). Diagnosis and treatment of adrenal insufficiency including adrenal crisis: A Japan Endocrine Society clinical practice guideline. Endocr. J..

[7] Bleicken B, Hahner S, Ventz M, Quinkler M (2010). Delayed diagnosis of adrenal insufficiency is common: A cross-sectional study in 216 patients. Am. J. Med. Sci..

[8] Katabami T, Tsukiyama H, Tanabe M, Matsuba R, Murakami M, Nishine A (2020). Development of a simple prediction model for adrenal crisis diagnosis. Sci. Rep..

[9] Barroso-Sousa R, Ott PA, Hodi FS, Kaiser UB, Tolaney SM, Min L (2018). Endocrine dysfunction induced by immune checkpoint inhibitors: Practical recommendations for diagnosis and clinical management. Cancer.

[10] Sekizaki T, Kameda H, Oba C, Cho KY, Nakamura A, Miyoshi H (2019). Nivolumab-induced hypophysitis causing secondary adrenal insufficiency after transient ACTH elevation. Endocr. J..

[11] Yamauchi I, Taura D, Hakata T, Fujita H, Okamoto K, Ueda Y (2021). Clinical features and thyroid dysfunction in adverse events involving the pituitary gland during PD-1 blockade therapy. Clin. Endocrinol. (Oxf.).

[12] Tahir SA, Gao J, Miura Y, Blando J, Tidwell RSS, Zhao H (2019). Autoimmune antibodies correlate with immune checkpoint therapy-induced toxicities. Proc. Natl. Acad. Sci. U. S. A..

[13] Kanie K, Iguchi G, Bando H, Urai S, Shichi H, Fujita Y (2021). Mechanistic insights into immune checkpoint inhibitor-related hypophysitis: A form of paraneoplastic syndrome. Cancer Immunol. Immunother..

[14] Inaba H, Ariyasu H, Iwakura H, Ueda Y, Kurimoto C, Uraki S (2019). Comparative analysis of human leucocyte antigen between idiopathic and anti-PD-1 antibody induced isolated adrenocorticotropic hormone deficiency: A pilot study. Clin. Endocrinol. (Oxf.).

[15] Yano S, Ashida K, Sakamoto R, Sakaguchi C, Ogata M, Maruyama K (2020). Human leucocyte antigen DR15, a possible predictive marker for immune checkpoint inhibitor-induced secondary adrenal insufficiency. Eur. J. Cancer.

[16] Ariyasu R, Horiike A, Yoshizawa T, Dotsu Y, Koyama J, Saiki M (2017). Adrenal insufficiency related to anti-programmed death-1 therapy. Anticancer Res..

[17] Cho KY, Miyoshi H, Nakamura A, Kurita T, Atsumi T (2017). Hyponatremia can be a powerful predictor of the development of isolated ACTH deficiency associated with nivolumab treatment. Endocr. J..

[18] Lupi I, Brancatella A, Cosottini M, Viola N, Lanzolla G, Sgrò D (2019). Clinical heterogeneity of hypophysitis secondary to PD-1/PD-L1 blockade: Insights from four cases. Endocrinol. Diabetes Metab. Case Rep..

[19] Reynolds RM, Seckl JR (2005). Hyponatraemia for the clinical endocrinologist. Clin. Endocrinol. (Oxf.).

[20] Miljic D, Doknic M, Stojanovic M, Nikolic-Djurovic M, Petakov M, Popovic V (2017). Impact of etiology, age and gender on onset and severity of hyponatremia in patients with hypopituitarism: Retrospective analysis in a specialised endocrine unit. Endocrine.

[21] Ferreira L, Silva J, Garrido S, Bello C, Oliveira D, Simões H (2017). Adrenal Tumors Study Group of the Portuguese Society of Endocrinology. Primary adrenal insufficiency in adult population: A Portuguese multicentre study by the Adrenal Tumours Study Group. Endocr. Connect..

[22] Hu Q, Gou Y, Sun C, Ding W, Xu K, Gu B (2014). The prognostic value of C-reactive protein in renal cell carcinoma: A systematic review and meta-analysis. Urol. Oncol..

[23] Guo L, Liu S, Zhang S, Chen Q, Zhang M, Quan P (2015). C-reactive protein and risk of breast cancer: A systematic review and meta-analysis. Sci. Rep..

[24] Woo HD, Kim K, Kim J (2015). Association between preoperative C-reactive protein level and colorectal cancer survival: A meta-analysis. Cancer Causes Control.

[25] Fang Y, Xu C, Wu P, Zhang LH, Li DW, Sun JH (2017). Prognostic role of C-reactive protein in patients with nasopharyngeal carcinoma: A meta-analysis and literature review. Medicine (Baltimore).

[26] Thorn GW, Forsham PH, Prunty FTG, Hills AG (1948). A test for adrenal cortical insufficiency. JAMA.

[27] Beishuizen A, Vermes I, Hylkema BS, Haanen C (1999). Relative eosinophilia and functional adrenal insufficiency in critically ill patients. Lancet.

[28] Puar TH, Stikkelbroeck NM, Smans LC, Zelissen PM, Hermus AR (2016). Adrenal crisis: Still a deadly event in the 21st century. Am. J. Med..

[29] Wang SA (2019). The diagnostic work-up of hypereosinophilia. Pathobiology.

[30] Bernard-Tessier A, Jeanville P, Champiat S, Lazarovici J, Voisin AL, Mateus C (2017). Immune-related eosinophilia induced by anti-programmed death 1 or death-ligand 1 antibodies. Eur. J. Cancer.

[31] Scanvion Q, Béné J, Gautier S, Grandvuillemin A, Le Beller C, Chenaf C (2020). Moderate-to-severe eosinophilia induced by treatment with immune checkpoint inhibitors: 37 cases from a national reference center for hypereosinophilic syndromes and the French pharmacovigilance database. Oncoimmunology.

[32] Krishnan T, Tomita Y, Roberts-Thomson R (2020). A retrospective analysis of eosinophilia as a predictive marker of response and toxicity to cancer immunotherapy. Future Sci. OA.

[33] Diehl A, Yarchoan M, Hopkins A, Jaffee E, Grossman SA (2017). Relationships between lymphocyte counts and treatment-related toxicities and clinical responses in patients with solid tumors treated with PD-1 checkpoint inhibitors. Oncotarget.

[34] Nakamura Y, Tanaka R, Maruyama H, Ishitsuka Y, Okiyama N, Watanabe R (2019). Correlation between blood cell count and outcome of melanoma patients treated with anti-PD-1 antibodies. Jpn. J. Clin. Oncol..

[35] Simon SCS, Utikal J, Umansky V (2019). Opposing roles of eosinophils in cancer. Cancer Immunol. Immunother..

[36] Kobayashi T, Iwama S, Yasuda Y, Okada N, Okuji T, Ito M (2020). Pituitary dysfunction induced by immune checkpoint inhibitors is associated with better overall survival in both malignant melanoma and non-small cell lung carcinoma: A prospective study. J. Immunother. Cancer.

