# Supplementary material for: Eosinophil counts can be a predictive marker of immune checkpoint inhibitor-induced secondary adrenal insufficiency: a retrospective cohort study
Source: Sci Rep. 2022 Jan 25;12:1294. doi: 10.1038/s41598-022-05400-x (PMC8789805; doi:10.1038/s41598-022-05400-x)
Supplement: Supplementary file 1 — Supplementary Information. [file 41598_2022_5400_MOESM1_ESM.pdf]

Eosinophil counts can be a predictive marker of immune checkpoint inhibitor-induced secondary adrenal insufficiency: A retrospective cohort study

Shinobu Takayasu, Satoru Mizushiri, Yutaka Watanuki, Satoshi Yamagata, Mari Usutani, Yuki Nakada, Yuko Asari, Shingo Murasawa, Kazunori Kageyama, Makoto Daimon.

Supplementary Table 1

	n = 22
Age (y)	70.6 ± 9.2
Sex (n)	
Male	12
Female	10
Primary sites (n)	
Lung cancer	8 (36.3%)
Renal-urinary cancer	6 (27.3%)
Malignant melanoma	3 (13.6%)
Gastric cancer	3 (13.6%)
Head and neck cancer	1 (4.5%)
Endometrial cancer	1 (4.5%)
ICI class (n)	
PD-1	
Pem	10
Niv	8
PD-L1	
Atez	1
Dur	1
PD-1 > PD-L1	
Pem > Atez	1
CTLA-4 + PD-1	
Ipi + Niv	1
Day of diagnosis of thyroid irAEs (d)	42 (57.6)

Supplementary Table 1. Characteristics of patients with thyroid irAE.

Note: Age is expressed as average ± standard deviation at diagnosis. A > B indicates change in immune checkpoint inhibitor from A to B. 'Day' is the median (average) number of days from the first administration of ICI.

Abbreviation: Atez, atezolizumab; CTLA-4, cytotoxic T-lymphocyte-associated protein 4; Dur, durvalumab; ICI, immune checkpoint inhibitor; irAEs, immune-related adverse events, Ipi, ipilimumab; Niv, nivolumab; PD-1, programmed cell death-1; PD-L1, programmed death-ligand 1; Pem, pembrolizumab.

Supplementary Figure 1

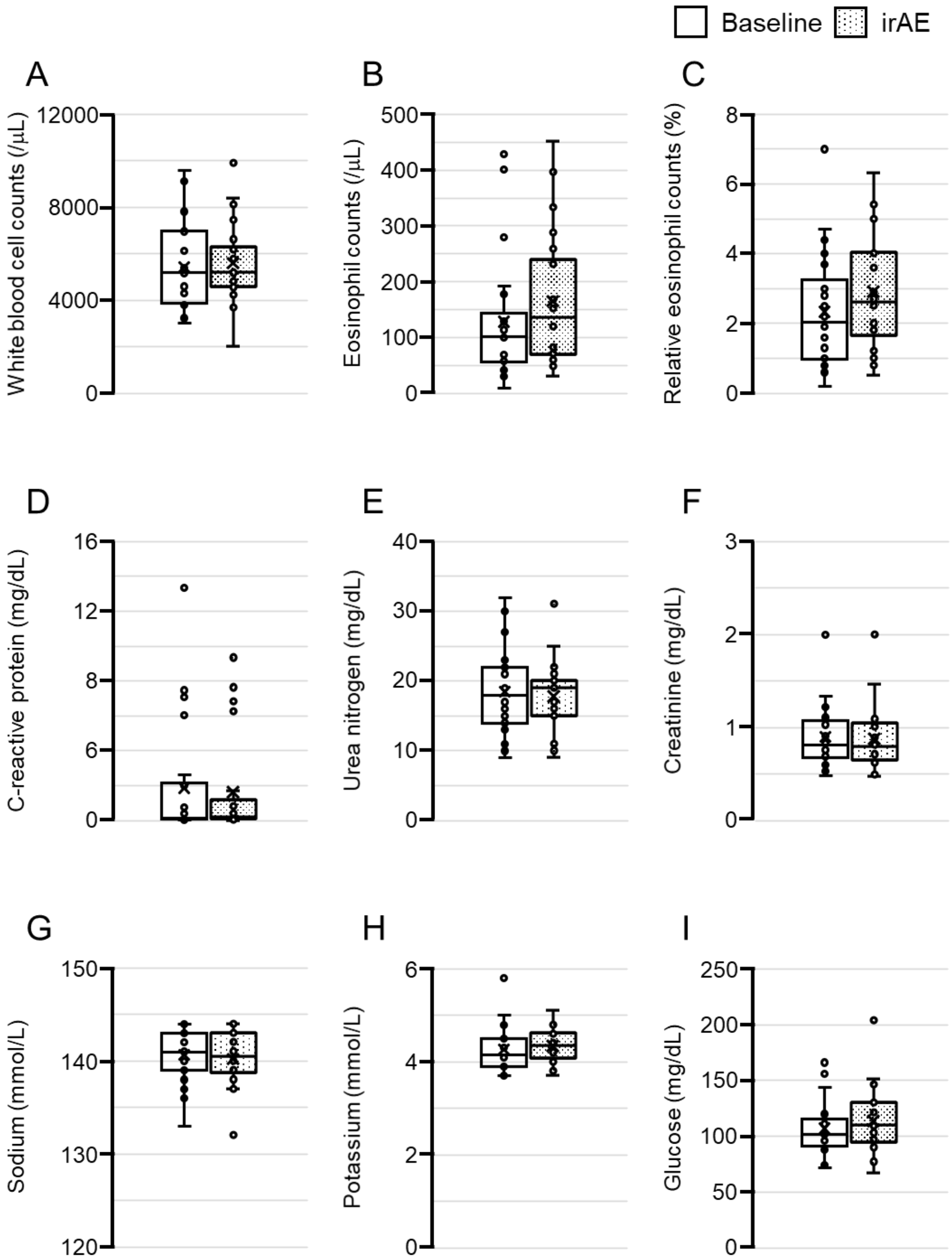

Supplemental Figure legend

Figure 1

Longitudinal data in the thyroid irAE group are shown by box plots. (A) White blood cell counts, (B) absolute eosinophil counts, (C) relative eosinophil counts, (D) C-reactive protein levels, (E) serum urea nitrogen levels, (F) serum creatinine levels, (G), serum sodium levels, (H) serum potassium levels, and (I) plasma glucose levels. ‘Baseline’ represents data before the first administration of ICIs. ‘Pre-onset’ represents data checked-up just before the patients experienced AI symptoms. ‘irAE’ represents data at the diagnosis of ICI-induced secondary AI. Statistical analyses were performed by Friedman's test corrected by Bonferroni's method.
